# The transplant iron score as a predictor of stem cell transplant survival

**DOI:** 10.1186/1756-8722-2-44

**Published:** 2009-10-24

**Authors:** Jonathan A Storey, Rebecca F Connor, Zachary T Lewis, David Hurd, Gregory Pomper, Yi K Keung, Manisha Grover, James Lovato, Suzy V Torti, Frank M Torti, István Molnár

**Affiliations:** 1 Department of Internal Medicine, Section on Hematology and Oncology, Wake Forest University School of Medicine, Winston-Salem, NC, USA; 2 Department of Pathology, Wake Forest University School of Medicine, Winston-Salem, NC, USA; 3 Department of Medicine, New York University Downtown Hospital, New York, NY, USA; 4 Department of Public Health Sciences, Wake Forest University School of Medicine, Winston-Salem, NC, USA; 5 Department of Biochemistry, Wake Forest University School of Medicine, Winston-Salem, NC, USA; 6 Department of Cancer Biology, Wake Forest University School of Medicine, Winston-Salem, NC, USA; 7 Comprehensive Cancer Center of Wake Forest University, Wake Forest University School of Medicine, Winston-Salem, NC, USA

## Abstract

Recent studies have suggested that the presence of iron overload prior to stem cell transplantation is associated with decreased survival. Within these studies, the criteria used to define iron overload have varied considerably. Given the lack of consensus regarding the definition of iron overload in the transplant setting, we sought to methodically examine iron status among transplant patients. We studied 78 consecutive patients at risk for transfusion-related iron overload (diagnoses included AML, ALL, MDS, and aplastic anemia) who received either autologous or allogeneic stem cell transplant. Multiple measures of iron status were collected prior to transplantation and examined for their association with survival. Using this data, three potentially prognostic iron measures were identified and incorporated into a rational and unified scoring system. The resulting Transplant Iron Score assigns a point for each of the following variables: (1) greater than 25 red cell units transfused prior to transplantation; (2) serum ferritin > 1000 ng/ml; and (3) a semi-quantitative bone marrow iron stain of 6+. In our cohort, the score (range 0 to 3) was more closely associated with survival than any available single iron parameter. In multivariate analysis, we observed an independent effect of iron overload on transplant survival (p = 0.01) primarily attributable to an increase in early treatment-related deaths (p = 0.02) and lethal infections. In subgroup analysis, the predictive power of the iron score was most pronounced among allogeneic transplant patients, where a high score (≥ 2) was associated with a 50% absolute decrease in survival at one year. In summary, our results lend further credence to the notion that iron overload prior to transplant is detrimental and suggest iron overload may predispose to a higher rate of lethal infections.

## Introduction

Long-standing iron overload can lead to heart and liver failure, resulting in premature death [1]. As our ability to treat iron overload improves, it is increasingly important to identify patients at risk for developing complications secondary to iron overload. Stem cell transplant patients are at risk for excess accumulation of iron resulting from repeated blood transfusions both before and during transplantation [2]. Because of this risk, it is recommended that transplant survivors with good long-term prognoses be assessed for iron overload [3]. Because iron overload has been perceived to be of primarily long term detriment, the measurement of iron status *prior *to transplant has not routinely been performed. However, recent evidence suggests that the determination of iron status before transplant has important prognostic implications [4-6].

Iron overload prior to transplantation was initially identified as a marker of poor prognosis in pediatric β-thalassemia patients [7]. Among those allogeneic transplant recipients, the presence of iron-induced portal fibrosis or hepatomegaly was associated with decreased survival. A later study by Altes et al. suggested that iron overload also adversely impacted those with hematologic malignancies [4]. In that study, very high levels of serum ferritin and transferrin saturation greater than 100% were used as surrogates for iron overload. Meanwhile, a larger study by Armand et al. defined iron overload based solely on serum ferritin, using the highest quartile for each disease type [6]. Using that definition of iron overload, a significant association with transplant survival was seen in patients with myelodysplastic syndrome (MDS) and acute myeloid leukemia (AML).

While each of these retrospective studies suggests that iron overload adversely affects transplant outcome, the clinical definition of iron overload varied considerably between studies. We set out to examine multiple measures of pre-transplant iron status with the goal of determining which marker(s) were most closely associated with clinical outcome following transplant. We chose to study patients at risk for transfusion related iron overload (diagnoses included acute leukemia, MDS, and aplastic anemia) undergoing either autologous or allogeneic transplant. Three measures related to transfusional iron overload were closely associated with transplant survival: (1) number of blood unit transfusions, (2) serum ferritin, and (3) bone marrow iron stores. These readily available measures were combined into a clinical scoring system termed the Transplant Iron Score.

The Transplant Iron Score showed a strong independent association with overall survival. Our findings further validate the detrimental impact of iron overload in the setting of stem cell transplantation and identify a potential mechanism of action.

## Methods

We evaluated 78 consecutive adult patients admitted to the Wake Forest transplant unit with a diagnosis of AML, MDS, acute lymphoblastic leukemia (ALL), or aplastic anemia. The included patients were all undergoing their first hematopoietic stem cell transplant between September 9, 1999 and March 19, 2004. The patient demographics and characteristics are summarized in Table 1. This study was approved by both the Protocol Review Committee of the Comprehensive Cancer Center of Wake Forest University and the Institutional Review Board of Wake Forest University School of Medicine.

**Table 1:** Patient characteristics

**Patient Characteristics**	**All Patients Number**	**High Iron Score number (percent)**	**Low Iron Score number (percent)**
Number	77	27	50
Median age	46	49	44
Sex			
Male	38	15 (56)	23 (46)
Female	39	12 (44)	27 (54)
Diagnosis			
AML	55	18 (67)	37 (74)
ALL	9	5 (19)	4 (8)
MDS	8	3 (11)	5 (10)
Aplastic anemia	5	1 (4)	4 (8)
Cytogenetics			
Favorable	3	2 (7)	1 (2)
Average	29	9 (33)	20 (40)
Poor	20	7 (26)	13 (26)
Disease state			
Non-proliferative	14	4 (15)	10 (20)
First remission	35	6 (22)	29 (58)
Second remission	17	9 (33)	8 (16)
No remission	11	8 (30)	3 (6)
Transplant type			
Autologous	31	8 (30)	23 (46)
Allogeneic	46	19 (70)	27 (54)
Matched related	27	9 (33)	18 (36)
Unrelated	19	10 (37)	9 (18)
Non-ablative	9	4 (15)	5 (10)

Values indicate the number of patients unless otherwise indicated. Percentages (%) may not add up to 100 due to rounding. A high iron score refers to a Transplant Iron Score of 2 or 3, while a low score represents a 0 or 1. AML indicates acute myeloid leukemia; ALL, acute lymphoblastic leukemia; MDS myelodysplastic syndrome; AA, aplastic anemia.

All serum samples were obtained upon admission to our bone marrow transplant unit, prior to the initiation of the preparative chemotherapy. Samples were continuously stored at -20°C, until measurements of iron parameters were performed. Serum ferritin levels were measured using a two-site chemiluminometric sandwich immunoassay (ADVIA Centaur^® ^Ferritin assay, Bayer Diagnostics, Tarrytown, NY). Transferrin saturation was calculated using the method by Huebers and Finch [8]. Serum levels of transferrin receptor (sTfR) were measured using a commercially available sandwich enzyme immunoassay (EIA) (Ramco Laboratories, Inc. Stafford, TX). C-reactive protein was measured using an enzyme-linked immunosorbent assay (high sensitivity) kit from American Laboratory Products Company, Windham, NH. The kit shows no cross-reactivity against albumin, lysozyme, alpha-1 antitrypsin and other acute phase proteins. Values for aspartate transaminase (AST), alanine transaminase (ALT), total bilirubin (TB), and the international normalized ratio (INR) for blood clotting time were obtained by review of the medical record. All recorded values were within 1 month of the admission date to our unit.

Bone marrow samples obtained within two months of transplantation were reviewed by two of the authors for specimen adequacy (I.M. and Z.L.), and representative sections of the patient's samples were stained with the Gomori's iron stain method [9]. Iron content of the bone marrow was graded by one of the authors (Z.L.) who was blinded to patients' laboratory and clinical parameters. Grading of marrow iron stores was scored according to previously published methods using a 0 to 6+ classification scheme described in detail by Gale et al [10]. Higher grades were associated with increased visible iron with a score of 6+ having large visible iron clumps that obscure cellular details. The number of packed red cell blood cell (pRBC) transfusions prior to transplantation was determined by blood bank records. The ejection fraction (EF) for each patient was based on the most recent pre-transplant echocardiogram or MUGA scan. All measures of EF were performed within 3 months of stem cell transplant. The highest quartile of total transaminases, TB, and INR among our study group was used as a surrogate for early liver dysfunction, while the lowest quartile of EFs was used to identify early pre-existing heart dysfunction.

Based on univariate quartile analysis, the three iron parameters with the strongest survival association were identified. Cutoff values for each parameter were determined independently using comparative statistics. Using this method, multiple pre-defined cutoffs were examined and compared for their association with survival. In order to maintain an adequate sample size on both sides of the cutoff, only values within the second and third quartile were considered. The selected cutoff values demonstrated the highest ability to discriminate survival based on a comparative analysis of hazard ratios.

Ultimately, these three iron parameters were incorporated into a unified scoring system. For each patient, a score was calculated by assigning a single point for each of the following: (1) serum ferritin ≥ 1,000 ng/mL, (2) greater than 25 transfused units of red cells, and (3) marrow iron stain of 6+. The sum of points, ranging between 0 and 3, was defined as the Transplant Iron Score. For missing data, no points were assigned. In the single patient where less than two parameters were available for scoring, the iron score was deemed indeterminate and was excluded from additional statistical analysis. The remaining 77 patients (99 percent) were included for analysis in our study. To allow further analysis within our study, a score of two or greater was deemed "high" and those patients were considered to have transfusion related iron overload. Meanwhile, a score of zero or one was classified as a "low" score. For purposes of comparing the Transplant Iron Score to other individual or combinations of iron parameters, each iron parameter was scaled be scored on a 0 to 3 scale. Using this approach, the individual iron parameters were divided into one of four quartile groups, similar to the four possible score groups defined by the Transplant Iron Score. Based on these groupings, a univariate relative risk of death was calculated for each iron parameter using hazard regression analysis.

Survival time was measured from the date of transplant to the date of death or last known follow-up. All data was censored as of July 1^st ^2007. The following clinical and demographic parameters were collected for statistical analysis: age at the time of transplant, gender, diagnosis, disease status (no remission, first remission, second remission), transplant type (autologous, related or unrelated allogeneic stem cell transplantation), and cytogenetic data for acute myeloid leukemia patients at the time of diagnosis. Cytogenetic information was grouped into poor, average and favorable categories based on the study by Byrd et al [11]. The specific cause of death for each patient was determined by chart review and categorized into disease related mortality, treatment related mortality, or not determined. All deaths following documented disease relapse were categorized as disease related mortality. Deaths attributable to treatment related mortality were further subdivided into deaths resulting from infection, graft-versus-host disease (GVHD), or veno-occlusive disease (VOD) of the liver. Documented infectious deaths were defined by the presence of a positive culture. Cases of suspected lethal infection met strict criteria for sepsis including radiologic imaging consistent with infection [12]. Kaplan-Meier curves were used to estimate median survival and overall survival differences. Cox proportional hazards regression was used to perform multivariate analysis. Results were considered significant when p-values were less than 0.05.

## Results

Multiple measures related to iron homeostasis were collected prior to stem cell transplant and are listed in Table 2. The individual iron parameter most closely associated with overall survival was the transfusion total, defined as the number of red cell units received prior to transplant. For each increase in quartile (e.g. 50^th ^to 75^th ^quartile) of transfused blood, the risk of death following transplant increased by a factor of 1.4. Serum ferritin was also significantly associated with transplant survival (p = 0.02), while the marrow iron stain showed a strong trend towards statistical significance (p = 0.08). Though not significant by quartile analysis, a bone marrow iron stain score of +6 was significantly associated with increased mortality (p = 0.04). The number of patients above the cutoff for transfusion number, ferritin, and iron stain were 30, 41, and 7, respectively. The Transplant Iron Score, when compared with individual and a combination of iron parameters, was most closely associated with survival (p = 0.0006). The risk of death nearly doubled with each point increase of the Transplant Iron Score.

**Table 2:** Association of iron parameters on transplant survival

**Iron Parameter(s)**	**Median**	**Relative Risk**	**p-value**	**Included in iron score**
Blood Transfusions (units)	22	1.40	0.007	YES
Serum Ferritin (ng/mL)	1103	1.36	0.02	YES
Marrow Iron Stain Grade^10^	4+	1.34	0.08	YES
Transferrin	193	0.77	0.11	No
Transferrin Receptor	6.1	0.80	0.12	No
Serum Iron (mcg/dL)	90	0.91	0.50	No
Transferrin Saturation (%)	30	1.08	0.54	No
Ferritin + Transfusions*	--	1.43	0.002	--
Ferritin + Iron Stain*	--	1.49	0.010	--
Transfusion + Iron Stain*	--	1.58	0.003	--
Transplant Iron Score*	**--**	**1.77**	**0.0006**	**--**

The relative risk represents the relative risk of death associated with each incremental increase in quartile (e.g. 50^th ^to 75^th ^quartile) among the iron parameters. The following cutoff values were used to assign patients to the various groupings: (1) serum ferritin ≥ 1,000 ng/mL, (2) greater than 25 transfused units of red cells, and (3) bone marrow iron stain of 6+. The Transplant Iron Score is calculated by assigning patients one point for each of the values above the cutoff.

*The calculated relative risks were scaled (i.e. scored on a 0-3 scale) to allow comparisons to the individual iron quartiles.

**Figure 1:**
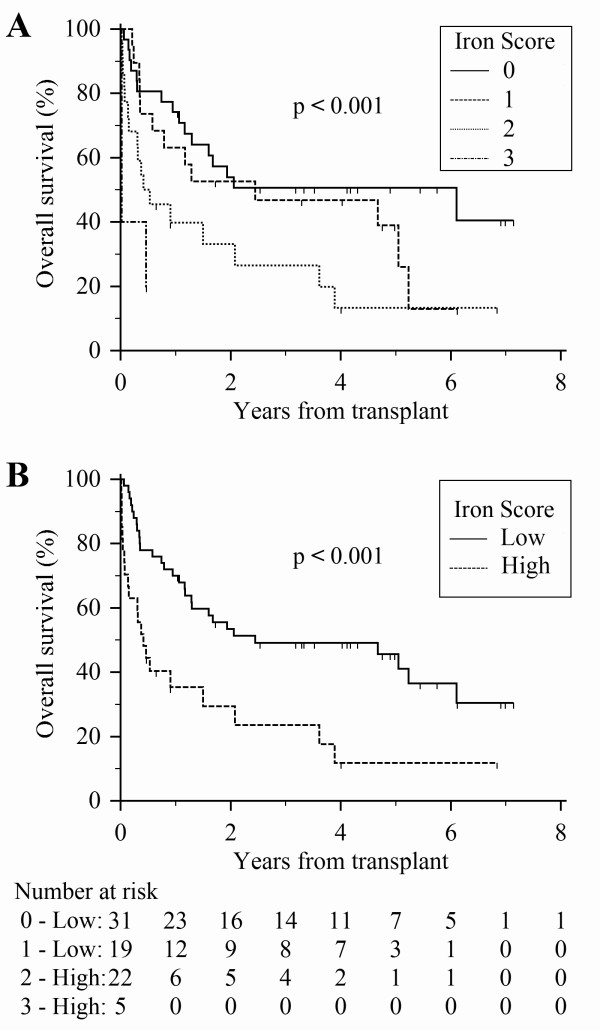
**Overall survival stratified by the Transplant Iron Score**. Patients were stratified based on the calculated Transplant Iron Score. (A) Score of 0 to 3 as defined by the scoring system. (B) High score (≥ 2) versus a low score (0 or 1). A number at risk table is included for each score group.

Trend analysis further supported that higher Transplant Iron Scores were associated with decreased overall survival (Figure 1A, p = 0.0003 by log-rank trend). The median survival for patients with no evidence of iron overload (score of 0) was estimated at over 6 years. Patients with higher scores had lower median survival times: 2.4 years for a score of 1; 6.5 months for a score of 2; and 8 days in those patients with a score of 3. The 27 patients (35%) with a high score had a substantially lower median survival of 5.0 months compared to 29.3 months in those with a low score (Figure 1B). The unadjusted hazard ratio associated with a high score was 2.60 (95% CI of 1.47 to 4.61). Our sample size did not allow us to perform rigorous subgroup analyses by disease type, however we did note that ALL (p = 0.02), AML (p = 0.06), and MDS (p = 0.004) patients with a high iron score exhibited a significant decrease in survival. Iron overload also resulted in decreased survival among aplastic anemia patients, however this did not reach statistical significance (p = 0.35).

**Figure 2:**
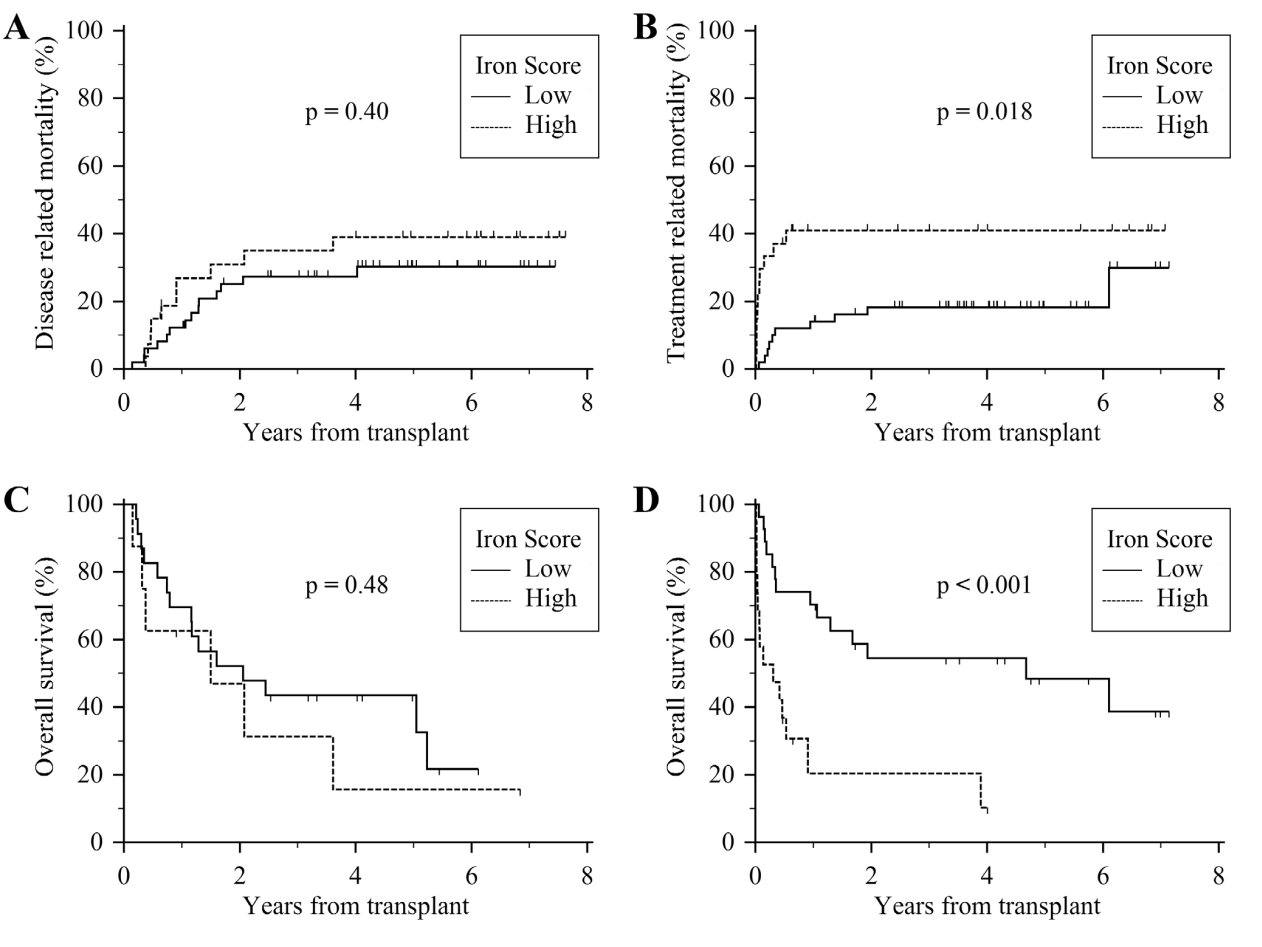
**Transplant outcomes stratified by the Transplant Iron Score**. (A) Disease related mortality for all patients. (B) Treatment related mortality for all patients. (C) Overall survival of autologous transplant patients. (D) Overall survival of allogeneic transplant patients.

The increase in mortality associated with iron overload resulted primarily from early deaths. In the first six months following transplant, 56% of those with a high iron score had died as compared to 22% among those with a low score (Figure 1B). This equates to a 34% absolute risk associated with transfusion related iron overload within the first six months. Subsequently, mortality rates were nearly identical between groups after the six-month time point. The difference in early survival was primarily due to an increased number of treatment related deaths (p = 0.018) (Figure 2B). Meanwhile, iron overload was not associated with a significant increase in relapse rate (p = 0.84) or disease related mortality (p = 0.40). The effect of iron overload was most pronounced among the patients undergoing allogeneic transplantation (p < 0.001) with a 50% absolute mortality difference at one year. Additionally, of the 19 allogeneic transplant patients with a high iron score, only two patients survived more than three years after transplant.

We further examined the cause of death among the 20 patients that died as a result of treatment related complications. The majority of these patients (55%) had either documented (5 patients) or suspected (6 patients) lethal infection. Furthermore, the rate of infection related mortality was disproportionately high among those with a high iron score (26%) as compared to those with a low score (8%) (p = 0.04 by Fisher's exact test). Clinical information regarding the 7 patients with a high iron score with infection-related mortality is detailed in Table 3. Rates of graft-versus-host disease (GVHD) and veno-occlusive disease (VOD) of the liver were statistically similar between groups (p = 0.9 and p = 0.3 by Fisher's exact test, respectively).

**Table 3:** Clinical characteristics of the patients with a high iron score and treatment-related death AML indicates acute myeloid leukemia

**Demographics**	**Disease**	**Donor**	**Conditioning**	**Iron Score (pRBCs)**	**Survival**	**Cause of Death**
53 y/o male	ALL (CR2)	MRD	Cytoxan/TBI	2 (45)	16 days	Clostridial sepsis
24 y/o female	AML (CR1)	MUD	Busulfan/Cytoxan	2 (42)	26 days	Septic shock (culture negative)
48 y/o female	AML (CR2)	MRD	Cytoxan/TBI	3 (38)	8 days	Pneumonia/ARDS
62 y/o female	Refractory AML	MRD	Non-ablative	2 (43)	50 days	CMV pneumonia
50 y/o female	MDS	MUD	Cytoxan/TBI	3 (35)	6 days	Pneumonia/ARDS
32 y/o male	AML (CR1)	MRD	Cytoxan/TBI	2 (58)	13 days	Septic shock (culture negative)
49 y/o female	Aplastic anemia	MUD	Cytoxan/TBI	2 (30)	27 days	Gram-negative sepsis

ALL, acute lymphoblastic leukemia; MDS myelodysplastic syndrome; MRD matched related donor; MUD matched unrelated donor; TBI total body irradiation; ARDS acute respiratory distress syndrome.

Multivariate analysis was used to establish whether the Transplant Iron Score was independently associated with transplant survival. Covariables included established predictors of transplant outcome, such as age, gender, donor-type, and remission status. In addition to standard transplant risk factors, an inflammatory marker (C-reactive protein) was included along with measures of end-organ damage (transaminase levels, INR, TB, and EF). Because frank organ failure is not likely to be present in eligible transplant patients, quartiles were used in the evaluation of end-organ damage in an attempt to identify early organ damage (i.e. mild transaminitis or a low-normal ejection fraction). The Transplant Iron Score had a significant independent effect on overall survival (p = 0.01) (Table 4). Among the subgroup of patients with AML, cytogenetic data (a marker for aggressive disease) did not influence the significance of this finding when added to the multivariate model. Furthermore, the iron score was independently associated with treatment related mortality (p = 0.04), infection related mortality (p = 0.02), and allogeneic transplant mortality (p = 0.03). When analyzing the subgroup of allogeneic transplant patients, a statistically significant difference in treatment related mortality (p = 0.01) and infection related mortality (p = 0.03) was maintained.

**Table 4:** Multivariate analysis of prognostic factors for stem cell transplant survival

**Potential Risk Factors**	**Covariables**	**Hazard Ratio**	**95% CI**	**p-value**
Iron Overload	Iron Score (0-3)	1.8	1.1 to 2.7	0.01
BMT Risk Factors				
Age	<40, 40s, 50s, 60s	1.5	0.9 to 2.3	0.08
Gender	Male	0.8	0.4 to 1.6	0.54
Donor-Type	Auto, Sibling, MURD	1.6	0.9 to 2.7	0.06
Remission Status	CR1, CR2, No remission	1.1	0.7 to 1.7	0.70
End-Organ Damage				
Heart Damage	Ejection Fraction	1.4	1.0 to 1.9	0.03
Liver synthetic function	INR	1.3	0.9 to 1.9	0.10
Hepatocellular damage	AST + ALT	0.8	0.6 to 1.2	0.32
Liver obstruction	Total Bilirubin	0.8	0.6 to 1.1	0.25
Inflammation	C-reactive protein	1.0	0.7 to 1.4	0.97

Potential risk factors were divided into ordered categorical variables when appropriate. The ejection fraction, INR, total transaminases, bilirubin, and C-reactive protein were categorized based on quartiles.

## Discussion

Estimating systemic iron stores in stem cell transplant patients is challenging. Serum ferritin has frequently been used as an estimate of systemic iron stores, but is prone to false elevation in the setting of inflammation and malignancy [13]. Other blood markers, such as transferrin saturation and soluble transferrin receptor, have proven to be even less successful in establishing the diagnosis of iron overload [14,15]. Non-invasive imaging techniques, such as T2* MRI, show promise for determining tissue iron stores but have not been extensively studied in transplant patients [16]. The current "gold standard" for assessing systemic iron overload remains dependent on liver biopsy [17]. However, invasive procedures are often not practical in patients awaiting stem cell transplant, as thrombocytopenia and neutropenia are common. Because of these difficulties, there is no consensus on how to best determine iron status in the transplant setting [18].

We identified three clinical markers of iron overload that were associated with decreased survival: (1) transfusion burden, (2) serum ferritin, and (3) bone marrow iron stores. Intuitively, each of these is a marker of transfusion related iron overload, and all have been used separately to estimate iron overload in transplant and non-transplant studies [5,6,19]. Each marker we identified also has the advantage of being readily available in the clinical setting, as exemplified by the high availability within our study.

In comparison to individual iron parameters, the Transplant Iron Score was more closely associated with transplant outcomes. Specifically, the iron score was more closely associated with survival than ferritin quartiles, which have previously been used to estimate pre-transplant iron overload [6]. Additionally, the iron score identified 35 percent of our study patients as having a "high" score, whereas quartiles by definition only identify the highest 25 percent. This suggests the Transplant Iron Score may be simultaneously more accurate and more inclusive than other proposed markers of iron overload in the transplant setting. Further evidence of the potential power of the iron score was seen in multivariate analysis, where the iron score maintained significance while controlling for other risk factors.

Using the Transplant Iron Score, we investigated the mechanism by which iron overload influences transplant survival. Classically, excess iron accumulates over decades resulting in progressive heart and liver dysfunction and eventually leading to premature death [1]. In contrast, our results demonstrate that iron overload at the time of transplant results in early mortality, and suggest that this process is not dependent on end-organ damage. Also differing from "classic" iron overload, our data suggests that a relatively low systemic iron burden is sufficient to substantially alter transplant survival. In adults, transfusion with more than 100 units of blood is generally required prior to clinical evidence of iron overload [20,21]. Meanwhile, even our patients with a high iron score had only 46 units of pRBCs on average. Taken together, our results suggest that iron overload in the transplant setting influences mortality by an alternate mechanism of action, differing from the classic model of chronic free-radical induced organ damage. Interestingly, the degree of iron overload necessary to impact transplant survival appears to be sub-clinical, underscoring the need for a more sensitive clinical marker of iron such as the Transplant Iron Score.

To further explore the mechanism by which iron overload influences survival, we closely examined the cause of death for each of the transplant recipients. We observed that treatment related mortality occurred more frequently in those patients with a high iron score. The majority of these deaths resulted from infection, thereby suggesting that lethal infection is the dominant mechanism by which iron overload influences transplant survival. While it has been suggested that iron overload predisposes to infection [4,5,22], to our knowledge, this is the first report showing an independent association between iron overload and infection related mortality.

In addition to adding insight into the mechanism of action of transplant iron overload, our study also helps to define its clinical applicability. Specifically, our study simultaneously compares the impact of iron overload in both the autologous and allogeneic transplant setting. Using the Transplant Iron Score to define iron overload in both groups, we found allogeneic transplant patients to be at a disproportionately high risk of death associated with iron overload. Our data suggests that iron overload as a prognostic marker may be limited to, or at least more pronounced in, patients undergoing allogeneic stem cell transplant.

We acknowledge the limitations inherent in our small single-institution study and believe that validation of the Transplant Iron Score is necessary prior to its incorporation into clinical practice. Nevertheless, our results strongly support the notion that iron overload prior to transplant is detrimental and provide rationale to study chelation therapy within the transplant setting. Typically, there is only a small window of opportunity between when a patient is identified as needing transplantation and when the transplant is undertaken. If patients with iron overload are detected early in this process, it is conceivable that iron chelation could minimize the negative impact of iron overload on transplant survival. This exciting possibility merits study in a prospective randomized fashion.

## Competing interests

The authors declare that they have no competing interests.

## Authors' contributions

JAS co-authored the manuscript, participated in the study design, collected clinical data, and performed the statistical analysis. RFC co-authored the manuscript, collected clinical data, and performed the laboratory testing. ZL performed the iron stain grading and provided pathology expertise. DH provided the blood samples and participated in the design of the study. GP provided data and expertise from our blood bank. YKK participated in the design of the study. MG assisted with data collection. JL participated in the statistical design. SVT participated in design of the study and assisted with proofreading. FMT participated in the design and coordination of the study. IM conceived of the study, and participated in its design and coordination.
